# Outcomes of Wild Type and TP53‐Mutated B Cell Malignancy Patients Receiving CAR‐T Cell Therapy: A Systematic Review and Meta‐Analysis

**DOI:** 10.1111/jcmm.70818

**Published:** 2025-09-22

**Authors:** Wenxin Qi, Yuqi Zhang, Xiaoyu Hao, Ping Yang, Jing Wang, Chaoling Wu, Weilong Zhang, Hongmei Jing

**Affiliations:** ^1^ Department of Hematology, Lymphoma Research Center Peking University Third Hospital Beijing China

**Keywords:** B cell malignancies, CAR‐T therapy, dual targeting CAR‐T therapy, therapeutic outcome, TP53 mutation

## Abstract

P53 mutation (TP53m) is a common intrinsic factor involved in relapsed or refractory (R/R) B cell malignancies that associates with treatment resistance. As a novel immunotherapy, CAR‐T has been increasingly applied in TP53m B cell malignancies, yet whether it can overcome the poor outcome of the TP53m population is controversial. We searched MEDLINE and EMBASE to identify population‐based cohort studies that evaluated the CAR‐T treatment outcomes between wild type and TP53m patients in B cell malignancies. Meta‐analysis on their complete response (CR), partial response (PR), overall response rate (ORR), progression‐free survival (PFS) and overall survival (OS) was carried out and pooled risk ratios (RR) or hazard ratios (HR) were estimated. A total of 10 eligible studies reporting 848 patients with B cell malignancies from wild type and TP53m groups receiving CAR‐T therapy were selected. The CR and ORR were comparable in both wild type and TP53m patients either with B cell lymphoma or leukaemia (all *p* > 0.05). However, the TP53m group was associated with shorter PFS and OS in both diseases (all *p* < 0.05). In traditional single targeting CAR‐T therapy, the PFS and OS were shorter in the TP53m group than in the wild type group (all *p* < 0.05). In contrast, the former outcomes of the wild type and TP53m groups were comparable when receiving dual‐targeting CAR‐T treatment (all *p* > 0.05). Though the CR and ORR of wild type and TP53m groups were similar, the PFS and OS of B cell malignancy patients bearing TP53m were inferior to wild type patients receiving CAR‐T cell treatment. Notably, the CR, PFS and OS of wild type and TP53m groups exhibit the same therapeutic effect via CD19/22 CAR‐T cocktail therapy. In other words, the poor prognosis of TP53m patients may be overcome by double targeting CAR‐T mode.

## 1 Introduction

B cell malignancies are a group of B cell oriented haematological malignancies with high clinical and biological heterogeneity, including B cell lymphoma and leukaemia. With the heterogeneity of the disease evolving, traditional frontline immunochemotherapy leading to failure is not rare. The following salvage high‐dose chemotherapy (HDT) with subsequent autologous stem cell transplantation (ASCT) is promising for these relapsed or refractory patients and has potential for long‐term event‐free survival (EFS) [1]. Yet the real‐world data is much disappointing. In the case of diffuse large B cell lymphoma (DLBCL), the most common non‐Hodgkin's lymphoma, studies suggest that only 50% of relapsed or refractory patients are candidates for HDT and ASCT. Since the introduction of rituximab, the expected rate of 3‐year EFS after ASCT is only approximately 20% [2].

Over the past two decades, improved insights into B cell malignancies, in terms of epidemiology, prognostic factors, and biologic heterogeneity, have led to the development of new therapeutic approaches [3]. CAR‐T cell therapy is an innovative therapeutic concept and represents a game‐changing therapeutic option by supporting the patient's own immune system to kill the tumour cells. It has resulted in unprecedented response rates in the R/R population. As ever‐increasing related clinical trials are carried out, a total of six CAR‐T cell‐based products have been approved for the treatment of B cell malignancies, including tisagenlecleucel (tisa‐cel) and axicabtagene ciloleucel (axi‐cel), with an overall CR rate of 80%–90% in R/R B‐ALL patients and 50% in R/R B‐cell lymphoma patients [4]. Although CD19 CAR‐T cell therapy has achieved amazing overall CR rates in patients with R/R B‐ALL and B cell lymphoma, 40%–60% of CR patients eventually experience relapse [5]. In axi‐cel‐treated patients with R/R large B cell lymphoma (LBCL), a 1‐year EFS rate of 43% (95% CI, 33%–52%) and a 2‐year EFS rate of 38% (95% CI, 28%–47%) were reported [6]. Likewise, in a cohort of 211 B‐ALL patients receiving tisa‐cel treatment, the 1‐year EFS rate was 52.4% (95% CI, 43.4%–60.7%) [7]. Studies suggest one of the resistances may be associated with diminished antigen density and have demonstrated that effective CAR‐T cell responses require high target antigen expression density [8, 9]. To optimise the therapeutic effect, innovative dual targeting CAR‐T therapy was developed. Either by sequential infusion of CARs against different antigen targets or fusing antigen binding moieties to one single CAR stem may solve the target loss problem. For example, sequential infusion of BCMA CAR‐T and CD19 CAR‐T has been proved safer and more effective than BCMA CAR‐T alone in multiple myeloma patients [10]. Wang et al. [11] achieved longer immune persistence and OS than CD19 CAR‐T by targeting CD19/CD22 tandem Bi‐CAR‐T in the treatment of B‐ALL. However, current real‐world data on whether dual targeting CAR‐T products own a significant advantage in treatinghighly aggressive B cell malignancies compared to traditional single target CAR‐T therapy is relatively rare [12, 13]. Unveiling the resistance mechanism seems the key to improving CAR‐T efficacy.

The intrinsic factors mark a fundamental aspect of relapse. Among them, TP53 mutation has been identified as a marker of poor prognosis and is often associated with therapeutic resistance. TP53 is known as a transcription factor that is, tightly regulated by post‐translational modifications to execute its diverse functions in tumour suppression [14]. TP53 mutation refers to genetic alterations in the TP53 gene; there are three main mutation types. Missense mutations are the most common, up to 80% of all mutations. The single amino acid change (e.g., R175H, R248Q) often causes loss of function or dominant‐negative effects. Nonsense or frame‐shift mutations refer to premature stop codons or truncated nonfunctional p53. Deletions or insertions disrupt protein structure or expression. In addition, TP53 mutation can occur in one allele (monoallelic) or both alleles (biallelic), with different implications for cancer development and progression. Monoallelic TP53 mutation means loss of mutation in one copy of the TP53 gene, leading to haploinsufficiency. Yet in biallelic TP53 mutation, both copies of TP53 are mutated, leading to complete loss of tumour suppressor function, which is typically found in advanced or high‐grade tumours [15]. These alterations may promote all cancer hallmarks such as sustaining proliferative signalling, deregulating cellular energetics, avoiding immune destruction, enabling replicative immortality, genome instability, and so on [16]. Its tumour‐suppressive nature makes TP53 seemingly undruggable. Correspondingly, it is regarded as a predictor for the efficacy of tumour treatments and is applied in patient stratification. Undoubtedly, multiple clinical pieces of evidence have demonstrated the negative role of TP53m in B cell malignancies [17, 18, 19, 20, 21, 22]. For example, an international collaborative study on 477 patients with DLBCL showed that TP53m has a worse effect on OS in DLBCL than TP53 naïve or mutants in other domains [23]. Similarly, in a cohort of 506 (wild type: *n* = 395, TP53m: *n* = 111) de novo DLBCL patients from multicentres receiving R‐CHOP therapy, the OS and PFS of the TP53m group were significantly inferior to the others [24]. However, its role in CAR‐T recipients is controversial. Wei et al. have compared the OS data of DLBCL between the wild type and TP53m patients, and the results suggest there is no statistical difference in OS within the two groups (*p* = 0.748). Similar results can be found in the studies of Shi et al. and Wang et al. [25, 26]. On the contrary, Shouval et al. and Wang et al. [27, 28] have demonstrated that CAR‐T therapy is less effective in the TP53 mutated population than in the naïve one.

There is no comparable evidence on the prognosis of TP53m B cell malignancy patients receiving CAR‐T treatment currently, while optimal therapy for this subpopulation is in urgent need due to its aggressive nature. As a potential weapon against blood cancer, a thorough understanding of CAR‐T therapy clinical outcomes in the TP53m patients over the past years may help establish an efficacy therapeutic strategy and improve their survival. Therefore, this article is endeavouring to collect multicenter clinical data to elucidate whether CAR‐T therapy can overcome the poor prognosis in B cell malignancy patients bearing TP53 mutation.

## 2 Methods

### 2.1 Search Strategy

A comprehensive search of the literature for the outcome of CAR‐T cell therapy in patients with B cell lymphoma and leukaemia was performed. The inclusion criteria followed Population, Interventions, Comparisons, Outcomes, and Study design (PICOS) principle. The population referred to B cell malignancy patients with TP53 mutation or deletion or wild type group. Intervention was receiving CAR‐T therapy. Outcomes included CR, ORR, PFS and OS. In terms of study design, randomised controlled trials (RCTs), non‐RCTs, prospective or retrospective observational studies and meeting reports were targeted (Table S1). Accordingly, preclinical/in vitro studies, editorials, commentaries, and case reports were excluded. MEDLINE and EMBASE were the main databases chosen. The detailed search strategy was in Table S2.

### 2.2 Sample Selection

An initial search result was acquired following the search strategies as mentioned above. We further filtered articles by skimming their titles and abstracts. Then full‐text publications of the studies that fulfilled the criteria in the title/abstract phase were retrieved. For intended or relevant literature, reference checking was also conducted to identify any missed publications. We also screened the publications reporting on the same study by comparing study authors, sample sizes and inclusion information to avoid double counting of outcomes in the final data set. Six studies were suspected of partial data overlapping in pairs, through quality assessment and sample size comparison, four studies were saved.

### 2.3 Outcome Definition

As the included literatures covered leukaemia and lymphoma, there were separate response assessment criteria. For B‐ALL, the CR was defined as bone marrow blasts less than 5%, platelets more than 100 × 10^9^/L, and neutrophils more than 1 × 10^9^/L. PR referred to all hematologic criteria of CR, yet the decrease of BM‐blast percentage from 5% to 25% and the decrease of pretreatment BM‐blast percentage by at least 50% [29]. The evaluation of CR in lymphoma was taken according to the Lugano classification criteria via marking radiological results. Specifically, CR was defined as PET‐CT score 1, 2, or 3 with or without a residual mass on 5PS or on CT, and target nodes or nodal masses must regress to less than 1.5 cm in longest diameter. PR referred to PET‐CT score 4 or 5 with reduced uptake compared with baseline and residual masses of any size. In a distinct study, ORR was collected as the combination of CR and PR (if both available) [30].

The survival outcomes of interest consisted of OS, which is the duration of time from either the date of diagnosis or the start of treatment for the disease to the death of patients, and PFS or leukaemia‐free survival (LFS), the duration of time after primary treatment the patient remained free of adverse outcomes, such as disease progression, local or distant recurrence, or death.

### 2.4 Quality Assessments

The Joanna‐Brigg's Institute assessment of study bias (JBI) was used to assess all non‐randomised controlled trials included; the criterions 1–10 were customised for this meta‐analysis. The Newcastle–Ottawa scale was applied to perform a quality assessment of all observative studies involved in the selection, comparability, and outcome. There were questions from each field listed aiding in assessment. A study can be awarded a maximum of one star for each numbered item. The more stars there are, the lower the risk of bias it indicates; conversely, fewer stars illustrate a higher risk of bias (Tables S3 and S4).

### 2.5 Statistics

Meta‐analysis of CR, ORR, PFS, and OS of qualified studies (with sufficient data) were conducted via Review Manager 5.3 version. Random‐effects model depended on the *p* value or *I* ^2^ was mostly applied to eliminate inter‐study heterogeneity. For certain studies without Hazards ratio (HR) between TP53m and wild type group on OS or PFS analysis yet offering the corresponding survival curve, Engauge Digitizer was used to acquire the survival data by matching the curve. Related HR was calculated through the formula from the supplementary Excel of the study from Tierney et al. [31]. *p* value of CR was calculated on IBM SPSS Statistics 27; the results were illustrated in a column graph on Graphpad Prism 9.5. Data were maintained in Microsoft Excel 2016 workbooks.

## 3 Results

### 3.1 Study Selection

The flow diagram of the selection process is shown in Figure S1. Systematic searches of MEDLINE and EMBASE identified 3831 studies for reports of CAR‐T cell therapy in the blood malignancy field. Through further screening, 10 articles, including 11 real‐world studies (published from 2020 to 2024), fulfilled the criteria and were selected for downstream analysis [1, 25, 26, 27, 28, 32, 33, 34, 35, 36] (Table 1). A total of 848 patients with B cell lymphoma (*n* = 487) or leukaemia (*n* = 361) treated with CAR‐T therapy were included. Wild type patients were 574 and TP53 mutated patients were 274. Of these identified studies, five were non‐RCTs and six were retrospective observational studies. There were four studies with 194 patients related to dual targeting CAR‐T therapy (wild type = 112, TP53m = 82), and specifically, three studies of B cell lymphoma and one of B‐ALL were included. Likewise, seven studies composed of two DLBCL, three mantle cell lymphoma (MCL) and two B‐ALL referred to single targeting CAR‐T treatment (*n* = 654, wild type = 462, TP53m = 192). With a median follow‐up time ranging from 11.3 to 35.6 months, the short‐term index CR and ORR or long‐term survival index PFS and OS were obtained either by being directly derived from the original study or calculated via raw data. Of note, to reduce bias and make inter‐study comparisons, the PFS and OS were all recorded as the one‐year ratio despite some longer outcomes being available. Details of those survival data could be found in Table 1.

**TABLE 1:** Summary of the sample size, survival outcomes, treatment, disease type and median following time in the 11 included studies.

		Author, year	Study type	Sample size			Wild type				TP53m				Treatment	Disease	Median following time (months)
				Total	Wild type	TP53m	CR (%)	PR	PFS	OS	CR (%)	PR	PFS	OS			
Dual targeting	1	Wei, 2022	Non‐RCT	65	34	31	17 (50.0%)	13	60.0% (1 year)	64.0% (1 year)	14 (45.2%)	13	56.0% (1 year)	64.0% (1 year)	CD19/22	DLBCL	13
	2	Na Wang, 2020	Non‐RCT	51	40	11	37 (92.5%)	2	60.0% (1 year)	60.0% (1 year)	11 (100.0%)	0	64.0% (1 year)	72.0% (1 year)	CD19/22	B‐ALL	16
	3	Na Wang, 2020	Non‐RCT	38	28	10	26 (92.9%)		42.0% (1 year)	53.0% (1 year)	7 (70.0%)		50.0% (1 year)	51.0% (1 year)	CD19/22	B‐NHL	13.6
	4	Li, 2023	Retrospective	40	10	30	9 (90.0%)		—	74.5% (1 year)	16 (53.6%)		—	46.5% (1 year)	CD19/22	B‐NHL	20.7
Single targeting	5	Hui Shi, 2023	Retrospective	80	40	40	21 (52.5%)		—	65.6% (1 year)	13 (32.5%)	27	—	50.0% (1 year)	CD19	DLBCL	17.2
	6	Pan, 2020	Retrospective	56	42	14	—	—	90.0% (1 year)	NA	6 (42.9%)	—	39% (1 year)	NA	CD19	B‐ALL	20.7
	7	Michael Wang, 2023	Non‐RCT	36	30	6	21 (70.0%)	9	—	NA	6 (100.0%)	0	NA	NA	CD19	MCL	11.3
	8	Shouval, 2022	Retrospective	82	52	30	33 (63.5%)	—	21.1 months	76.0% (1 year)	10 (33.3%)	—	34.0% (1 year)	44.0% (1 year)	CD19	DLBCL	21.1
	9	Zhang, 2022	Retrospective	254	221	33	206 (93.2%)	—	72.0% (1 year)	78.0% (1 year)	24 (72.7%)	—	48.0% (1 year)	55.0% (1 year)	CD19	B‐ALL	12
	10	Minson, 2024	Non‐RCT	20	12	8	9 (75.0%)	0	80.0% (1 year)	NA	7 (87.5%)	0	75.0% (1 year)	100.0% (1 year)	CD19	MCL	16.3
	11	Yucai Wang, 2023	Retrospective	126	65	61	57 (88.0%)	—	63.0% (1 year)	NA	44 (72.0%)	—	47.0% (1 year)	NA	CD19	MCL	35.6

### 3.2 Patients' Characteristics

The basic information of patients such as age, gender, disease stage, Eastern Cooperative Oncology Group (ECOG) status and bone marrow blasts was also investigated. Of the 11 studies included, the inter‐study age of subjects varied a lot; the average age of patients receiving dual targeting CAR‐T therapy was slightly younger than those under single targeting CAR‐T treatment. Both groups contained paediatric patients. There were 528 male and 285 female patients included, whose ratio was roughly 1.8:1. As for tumour burden, most patients included had relapsed or resistant B cell lymphoma with stage III or IV. Bone marrow blasts concentrated on 5%–20% in B‐ALL patients. With data available, the prior haematopoietic cell transplantation (HCT) ratio was 9.1%–55.0%, and the median previous line was roughly 3. The extra‐medullary tumour ratio was 19.7%–48.2%. The majority of patients were at their first refractory or relapse stage (58.9%–81.4%). Despite two studies not mentioning the specific TP53 mutation type of subjects, most included patients bore TP53 missense mutation, and the second common type was TP53 deletion (Table 2).

**TABLE 2:** Basic clinical characteristics of patients in the 11 included studies.

CAR‐T	Study name	Disease	Sample size	Age median (range)		Sex		ECOG/IPI status (%)		Stage (%)		BM blasts for morphology (%)	Prior HCT (%)	Previous line (%)	Disease burden/extra‐medullary disease (EMD)	First relapse/primary refractory	Main TP53 mutation type
				Wild type	TP53m	Male	Female	0–2	3–4	I–II	III–IV						
Dual targeting	Li, 2023	B‐NHL	40	8 (0–18)		33	7	—	—	0.0%	100%		—	—	—	81.4%	Missense mutation
	Wei, 2022	DLBCL	66	50 (17–69)	47 (29–63)	43	23	100.0%	0.0%	15.2%	84.8%		18.2%	≤ 3, 54.5%	Tumour mass ≥ 5 cm, 44.0%	76.0%	Missense mutation
														≥ 4, 45.5%			
	Na Wang, 2020	B‐NHL	38	47 (17–71)		22	16	59.5%	39.5%	10.5%	89.5%		15.8%	—	—	68.4%	Not mention
	Na Wang, 2020	B‐ALL	51	27 (9–62)		32	19	—	—	—	—	BM < 5% (25.5%) BM ≥ 5% (74.5%)	23.5%	—	—	66.7%	Not mention
Single targeting	Shouval, 2022	DLBCL	82	66 (58–72)		55	27	—	—	27.0%	72.0%		22.0%	≤ 3, 41.0%	—	—	Mutation
														4–5, 39.0%			
	Hui Shi, 2023	DLBCL	80	49 (13–79)		40	40	50.50%	49.5%	11.4%	88.6%		24.8%	—	EMD 47.6%	—	Missense mutation
	Yucai Wang, 2023	MCL	126	67 (34–89)		96	30	89.00%	11.0%	10.0%	90.0%		39 3%	3 (1–10)	Tumour mass > 10 cm, 14.0%	—	Mutation/deletion
	Zhang, 2022	B‐ALL	254	1–14 (56.6%) 15–61 (43.4%)		157	97	—	—	—	—	BM ≤ 20% (62.2%) BM > 20% (37.8%)	9.1%	Chemotherapy cycle > 10 29.1%	EMD 19.7%	—	Not mention
	Wang, Michael 2023	MCL	36	65 (38–79)		—	—	—	—	0.0%	100.0%		—	—	—	—	Deletion
	Minson, 2024	MCL	20	66 (41–47)		15	5	100.0%	0.0%	30.0%	70.0%		55.0%	—	—	—	Missense mutation
	Pan, 2020	B‐ALL	56	7 (1–18)		35	21	—	—	—	—		17.9%	—	EMD 48.2%	58.90%	Missense mutation

### 3.3 Intra‐Study and Inter‐Study Comparison of CR and ORR in Wild Type and TP53m B Cell Malignancy Patients Receiving CAR‐T Therapy

**FIGURE 1:**
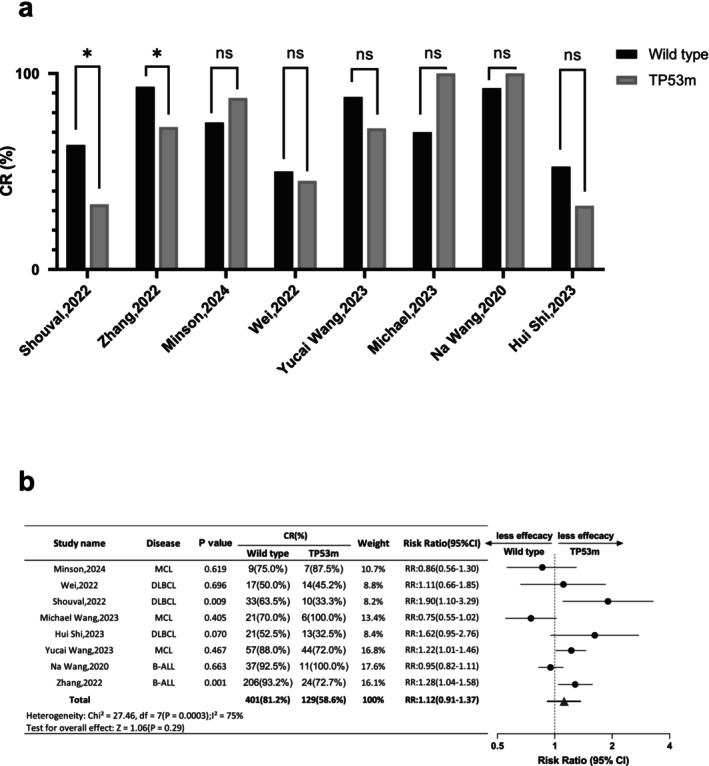
Intra‐study and inter‐study comparison of CR. (a) Column diagram illustrates comparison of CR between wild type and TP53m group in eight evaluable studies included. * Represents statistical significance, ns means no significance. (b) Forest plot of CR between wild type (TP53 naïve) and TP53m group of eight evaluable studies included.

**FIGURE 2:**
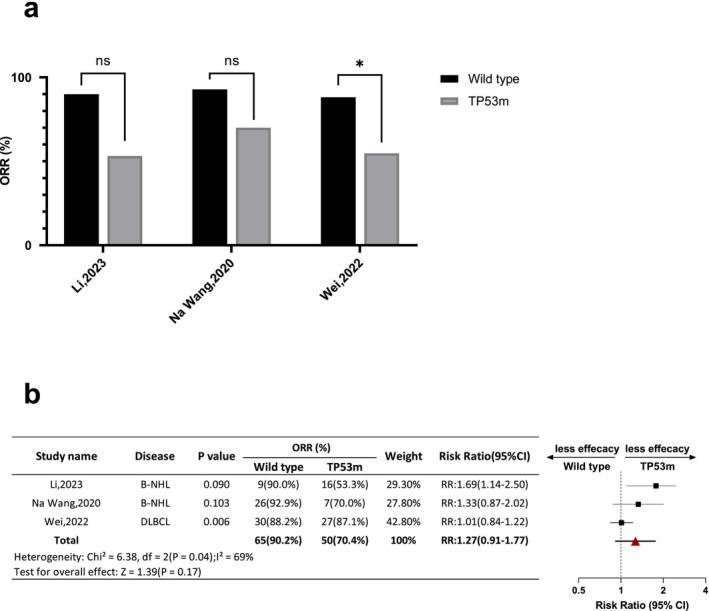
Intra‐study and inter‐study comparison of ORR. (a) Column diagram illustrates comparison of ORR between wild type and TP53m group in three evaluable studies included. * Represents statistical significance, ns means no significance. (b) Forest plot of ORR between wild type and TP53m group of three evaluable studies included.

According to data reported, there were eight studies (*n* = 714, wild type = 494, TP53m = 220) included eligible for CR analysis. Among which, the CR rate varied between 50.0% and 93.2% in the wild type group, and 32.5% and 100% in the TP53m group. In intra‐comparison, only two studies showed a statistical difference (Shouval, 2022 RR: 1.90 [95% CI: 1.10–3.29]; Zhang, 2022 RR: 1.28 [95% CI: 1.04–1.58]), while the others suggested the CR rate in the TP53m group was all comparable to the wild type group. The pooled RR of wild type groups vs. TP53m group was 1.12 (95% CI: 0.91–1.37) (Figure 1). Likewise, there were three studies (*n* = 143, wild type = 72, TP53m = 71) included evaluable for ORR analysis. The ORR rate varied between 88.2% and 92.9% in the wild type group, and 53.3% and 87.1% in the TP53m group. In intra‐comparison, one study showed a statistical difference of ORR within the two groups (Wei, 2022 RR: 1.61 [1.14–2.27]). The pooled RR of wild type groups vs. TP53m group was 1.27 (95% CI: 0.91–1.77) (Figure 2). Both CR and ORR were comparable in wild type and TP53m B cell malignancy patients receiving CAR‐T therapy.

### 3.4 Comparison of the Therapeutic Efficacy Between Wild Type and TP53m Patients With B Cell Lymphoma or B Cell Leukaemia Receiving CAR‐T Therapy

**FIGURE 3:**
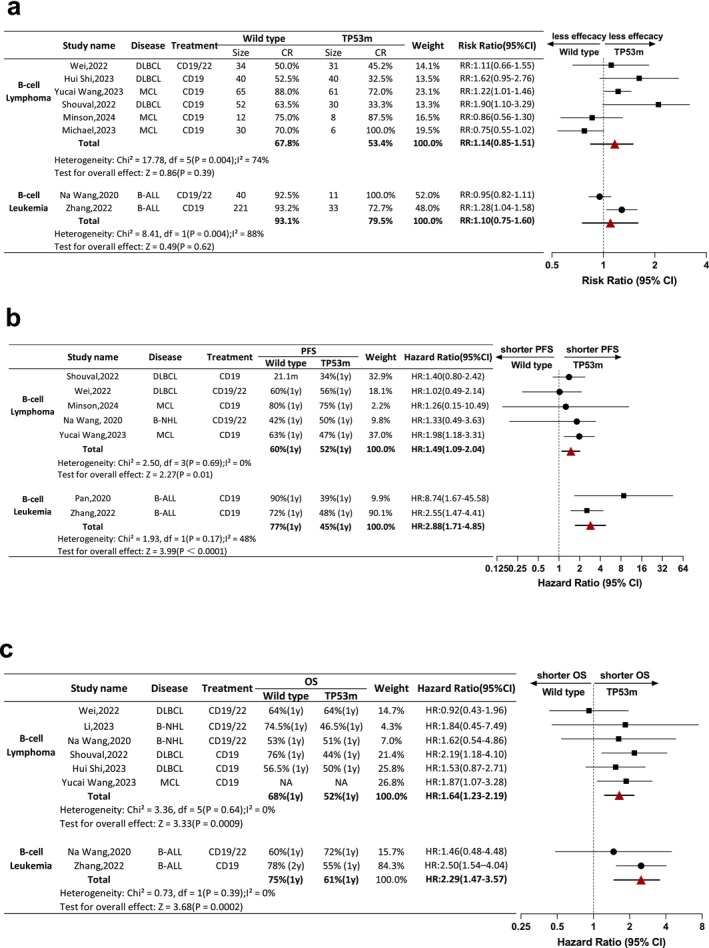
Comparison of CR, PFS and OS via B‐cell leukaemia and B‐cell lymphoma. (a) Outcome of CR of B‐cell leukaemia and B‐cell lymphoma population receiving CAR‐T therapy. (b and c) Outcome of PFS or OS of B‐cell leukaemia and B‐cell lymphoma population receiving CAR‐T therapy. CI, confidence interval; NA, not available, TP53m, TP53‐mutated.

Specifically, the reported data were compared regarding CAR‐T therapeutic efficacy (CR, PFS and OS) of wild‐type and TP53 m groups between B cell lymphoma and B cell leukaemia. Eight studies were included in the CR analysis. In the B cell lymphoma group, there were six studies with a total of 714 patients. Among which, the CR rate varied between 50.0% and 88.0% in the wild‐type group, and 32.5% and 100.0% in the TP53m group. The pooled CR was 67.8% in the wild‐type group and 53.4% in the TP53m group. The total RR of wild‐type groups vs. TP53m group was 1.14 (95% CI: 0.85–1.51). In contrast, there were two studies referred to B‐ALL, whose CR rate was 92.5% and 93.2% in the wild type group and 100.0% and 72.7% in the TP53 m group. The general CR in the wild type group was 93.1% and 79.5% in the TP53m group. The pooled RR of wild type groups vs. TP53m group was 1.10 (95% CI: 0.75–1.60) (Figure 3a). In other words, there was no statistical significance of CR between wild type and TP53m patients, whether with B cell lymphoma or leukaemia receiving CAR‐T therapy.

In the field of B cell lymphoma, there were five studies evaluable for PFS. Two on behalf of MCL suggested 80.0% of one‐year PFS in wild type group (*n* = 10) and 75% in TP53m group (*n* = 8, HR: 1.26 [95% CI: 0.15–10.49]), and 63.0% of one‐year PFS in wild type group (*n* = 65) and 47.0% in TP53m group (*n* = 61, HR: 1.98 [95% CI: 1.18–3.31]), respectively. Two studies of DLBCL suggested median PFS of 21.1 months in wild type group (*n* = 52) and one‐year PFS of 34.0% in TP53m group (*n* = 30, HR: 1.40 [95% CI: 0.80–2.42]), 60% of one‐year PFS in wild type group (*n* = 34) and 56% in TP53m group (*n* = 31, HR: 1.02 [95% CI: 0.49–2.14]), respectively. The pooled one‐year PFS ratio was 60.0% in wild type group and 52.0% in TP53m group. The overall HR of B‐NHL PFS in wild type groups vs. TP53m group was 1.49 (95% CI: 1.09–2.04). Two studies were included in B cell leukaemia group; the one‐year PFS in wild type group was 90.0% and 72.0%, and 39.0% and 48.0% in TP53m group. The pooled one‐year PFS rate was 77.0% in wild type group and 45.0% in TP53m group. The overall HR of B‐ALL PFS in wild type groups vs. TP53m group was 2.88 (95% CI: 1.71–4.85) (Figure 3b). Whether in B cell lymphoma or leukaemia, TP53m patients were at a PFS disadvantage compared to the wild type patients.

The one‐year OS rate of included studies was evaluated for the wild type and TP53m group therapeutic efficacy between B cell lymphoma and leukaemia. There were eight studies included (*n* = 710), six on behalf of the B cell lymphoma group (*n* = 428) and the rest represented B cell leukaemia (*n* = 282). In the setting of B cell lymphoma, the one‐year OS rate varied from 53.0% to 76.0% in wild type groups, and the estimated overall one‐year OS was 68.0% in this population. The intra‐study one‐year OS rate deviated from 44.0% to 66.0% in TP53m groups, and the pooled one‐year OS rate was 52.0%. One study (Yucai Wang, 2023) lacked a survival curve and the one‐year OS result was unavailable, yet the intra‐group HR of the wild type group vs. the TP53m group was provided as 1.87 (95% CI: 1.07–3.28). In comparison, the overall HR of the B cell lymphoma population OS in wild type groups vs. TP53m group was 1.64 (95% CI: 1.23–2.19). There were two studies estimating the OS for the B cell leukaemia population; the one‐year OS rate was 60.0% in the wild type group (*n* = 40) and 72.0% in the TP53m group (*n* = 11), HR: 1.46 (95% CI: 0.48–4.48). The other study illustrated a 78% one‐year OS rate in the wild type group (*n* = 207) and a 55% one‐year OS rate in the TP53m group (*n* = 24), HR: 2.50 (95% CI: 1.54–4.40). The pooled one‐year OS of the wild type group was 75.0% and 61.0% in the TP53m group. The overall HR in the B cell leukaemia population of wild type groups vs. TP53m groups was 2.29 (95% CI: 1.47–3.57) (Figure 3c). Likewise, TP53m patients had shorter PFS than wild type patients in both diseases.

**FIGURE 4:**
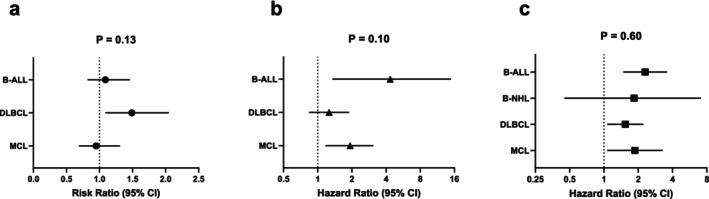
(a–c) Disease‐specific subgroup analysis of overall CR, PFS and OS. (a) Disease‐specific subgroup analysis of overall CR. (b and c) Disease‐specific subgroup analysis of overall PFS and OS.

The disease‐specific subgroup analysis of CR, PFS and OS of included patients were also produced (Figure 4a–c). There were three types of B cell malignancy (DLBCL, MCL and B‐ALL) included in overall CR analysis. Although the pooled RR of CR patients showed no statistical difference in spite of bearing TP53m or not. DLBCL exhibited poorer efficacy (RR: 1.49 [1.09, 2.05]) than MCL, which was consistent with clinical practice. The PFS and OS analysis revealed that pooled HR of DLBCL and B‐NHL were smaller than MCL and B‐ALL, which may be attributed to dual‐targeting CAR‐T treatment dominant in the former groups in contrast of single targeting CAR‐T therapy in latter groups. Of note, despite the subgroup difference, all *p* values in these analysis were over 0.05, suggesting no significant risk affect the pool effect size. More sensitivity analysis based on different comparison groups to classify influential factors were in Supporting Information (Figures S2–S4).

### 3.5 Comparison of the Therapeutic Efficacy Between Wild Type and TP53m of B Cell Malignancy Patients Receiving Dual Targeting and Single Targeting CAR‐T Treatment

**FIGURE 5:**
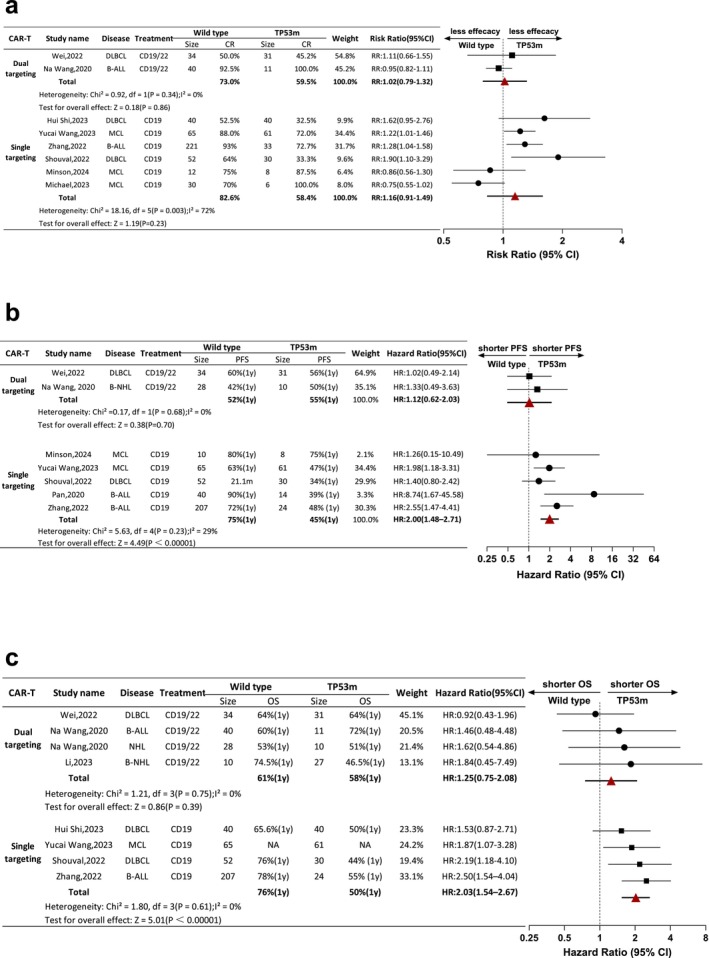
Comparison of CR, PFS and OS via dual targeting CAR‐T and single targeting CAR‐T therapy. (a) Outcome of CR of patients with B cell malignancy via CD19/22 vs. CD19 CAR‐T therapy. (b and c) Outcome of PFS/OS of patients with B cell malignancy via CD19/22 and CD19 CAR‐T therapy.

Therapeutic outcome of B cell malignancy patients via CAR‐T therapy with single or double targets was evaluated by CR, OS and PFS. There were eight studies (*n* = 714, wild type = 494, TP53m = 220) eligible for CR analysis. The duration to achieve CR in included studies varied from 30 to 90 days. Two studies were composed of dual targeting CAR‐T therapy group and the correlated CR rate is 50.0% and 92.5% in wild type group, 45.2% and 100.0% in TP53m group, respectively. The total CR was 73.0% versus 59.5%. In comparison, single targeting CAR‐T group comprised six studies, whose CR rate varied between 52.5% and 93.0% in wild type group and 32.5% and 100.0% in TP53m group. The total CR was 82.6% versus 58.4%. The forest plot illustrated similar therapeutic efficacy of TP53m group and wild type group whether in single or double targeting CAR‐T treatment (HR: 1.16 [95% CI: 0.91–1.49]; HR: 1.02 [95% CI: 0.79–1.32]) (Figure 5a).

Seven studies (*n* = 614, wild type = 436, TP53m = 178) were selected for PFS analysis. The dual targeting CAR‐T therapy group contained two studies; the one‐year PFS rate was 60.0% and 42.0% in the wild type group and 56.0% and 50.0% in the TP53m group, which were comparable in a forest plot (HR: 1.12 [95% CI: 0.62–2.03]). The pooled one‐year PFS rate was 52.0% in the wild type group and 55.0% in the TP53m group. In the single targeting CAR‐T group, there were five studies available. The one‐year PFS rate ranged from 63.0% to 90.0% in the wild type group and 34.0% to 75.0% in the TP53m group. The pooled one‐year PFS rates of 75.0% and 45.0% were found in the former two groups, respectively. Less therapeutic efficacy was demonstrated in the TP53m group receiving the CAR‐T product with a single target compared to that in the wild type group (HR: 2.00 [95% CI: 1.48–2.71]) (Figure 5b).

As for OS analysis, eight studies (*n* = 710, wild type = 476, TP53m = 234) were included. Among which, four studies belonged to the dual targeting CAR‐T group, the one‐year OS rate varied between 53.0% and 74.5% in the wild type group, and the pooled OS rate was 61.0% (95% CI: 0.52–0.57). In the TP53m group, the one‐year OS rate varied between 46.5% and 72.0%, with a pooled one‐year OS rate of 58.0%. Like CR and PFS mentioned above, the OS rate between the wild type and TP53 m groups was comparable as well (HR: 1.25 [95% CI: 0.75–2.08]). In the single targeting CAR‐T group, the one‐year OS rate varied between 65.6% and 78.0% in the wild type group, and the pooled OS rate was 76% (95% CI: 0.71–0.81). In comparison, the one‐year OS rate varied between 44.0% and 55.0% in the wild type group, whose pooled OS rate was 50.0% (95% CI: 0.40–0.59). The intergroup comparison result suggested the response to single targeting CAR‐T therapy of TP53m patients was less satisfactory than that of wild type patients (HR: 2.03 [95% CI: 1.54–2.67]) (Figure 5c).

## 4 Discussion

This meta‐analysis was undertaken to address a controversial clinical question: can CAR‐T therapy overcome the poor outcome of B cell malignancy patients with TP53 mutation? To our knowledge, the appearance of CAR‐T therapy has evolutionarily altered cancer treatment, especially in the haematological field, where it leads to remarkable, long‐term antitumour effects with exact target specificity. Nonetheless, either the concern for therapeutic failure, safety problems, or the expensive cost has still hampered its popularisation. The current research suggests that CAR‐T therapy exhibits powerful efficacy in R/R B cell lymphoma and leukaemia, where TP53m patients are an inescapable component [37]. The negative prognostic role of TP53m towards B cell malignancies is undoubtable. How to improve the outcomes of TP53m patients has perplexed doctors and scientists for several decades. Findings from this study confirm that patients with TP53m B‐ALL or lymphoma experience poorer outcomes compared to wild type patients despite receiving CAR‐T therapy in the longterm. The inter‐study comparison demonstrated that though the short‐term response index CR (RR: 1.12 [95% CI: 0.91–1.37]) and ORR (RR: 1.27 [95% CI: 0.91–1.77]) showed no statistical difference, yet the PFS and OS of B cell malignancy patients bearing TP53m were inferior to wild type patients receiving CAR‐T cell treatment in this cohort. In the setting of dual targeting or sequential CAR‐T therapy, the conclusion changes. Either the estimated CR, PFS, or OS in the wild type group and TP53m group of B cell malignancy patients exhibit similar therapeutic effects in response to CD19/22 CAR‐T therapy. By contrast, the population receiving CD19 CAR‐T therapy displays therapeutic differences whether on short‐term CR or long‐term indices PFS and OS. These findings indicate that dual CAR‐T treatment may overcome the detrimental prognosis in TP53m patients and makes no significant statistical difference in OS outcome compared to wild type patients of the studied disease.

TP53 mutations are known to predispose patients to second primary malignancies (SPM), especially in the context of genotoxic CAR‐T therapies. The underlining mechanisms may be attributed to the genetic integrity through DNA repair and genomic surveillance of T cells as the same as other somatic cells, either irreparable DNA damage or inaccurately repaired damage, such as mutations, duplications and inversions, could lead to oncogenesis. What's more, viral vector used to introduce CAR‐T cells to target may generise insertional mutagenesis and transcriptional deregulation as well [38]. Whether a SPM risk of CAR‐T therapy itself pose adverse effect on the poor prognosis of TP53m patients should be considered. According to data reported, SPM incidence is relatively low in blood malignancies (4.6%), making it difficult to analysis and draw a conclusive determination of risk in comparison of different treatment [38]. In a SPM meta‐analysis with 5571 lymphoma and myeloma patients, a subgroup meta‐analysis of randomised CAR‐T versus standard‐of‐care revealed a similar risk of SPM with either treatment strategy (*p* = 0.92), which does not indicate that SPM frequency is higher with CAR‐T therapy versus previous standard‐of‐care strategies [39]. Though larger cohort study may be required for validation, CAR‐T therapy is not taken as confounding factor in this analysis.

The highlighted finding in this meta‐analysis is dual targeting CAR‐T seemingly overcoming the poor outcome of TP53m patients and exerting potential therapeutic effects as the same as that in the wild type group. Coincidentally, a phase 1 trial of CD19/22 sequential infusion of CAR‐T therapy on recurrent or refractory B‐ALL was demonstrated with a CR rate of 95.2% and a two year OS of 77.6%, compared to patients who received CD19 CAR‐T therapy achieving a CR rate of 83.0% and a two year OS of 77.6%, respectively (*p* = 0.006; *p* = 0.0187) [40]. Also, the result of patients with high‐risk factors receiving tandem CD19/22 CAR‐T treatment had no statistical significance with cocktail trial patients whether in CR or long term OS (*p* = 0.135; *p* = 0.525), suggesting that sequential CD19/CD22 CAR T‐cell therapy obtains a better response than CD19 CAR T‐cell therapy and a similar response to tandem CD19/CD22 CAR T‐cell therapy [40]. The underpinning mechanism may be associated with cytokine release. Spiegel et al. [9] indicated CD19/22 CAR‐T led to decreased TNF‐α and IL‐2 secretion in comparison to CD19 CAR‐T therapy. The threshold for CAR‐T cell IL‐2 secretion has previously been shown to be higher than that of IFN‐γ20 and may better discriminate CAR efficacy [41].

In the setting of the CAR‐T era, despite the remarkable milestone it reaches, treatment failure still happens. One of the therapeutic resistances is attributed to intrinsic cellular factors like TP53 alteration [42]. Through RNA sequencing on samples of TP53m patients with LBCL treated with CD19 CAR‐T therapy, Shouval et al. [27] have summarised the resistance mechanism in four aspects: impairment of IFN signalling, dysregulation of the extrinsic apoptotic pathway, imposition of an immunosuppressive tumour microenviroment (TME), and downregulation of CD19. Some other studies also reveal the link between the resistance of TP53 alteration with negative antigen target in the context of CD19 CAR‐T therapy. Pan et al. [34] have reported a high rate of relapse occurred in TP53m R/R B‐ALL children treated with CD19 CAR‐T, where loss of CD19 was commonly observed in TP53m of this population. Li et al. and Sang et al. [43, 44] have demonstrated that co‐administration of CD19 and CD20 or CD22 could overcome antigen escape and prolong the survival of B‐ALL or DLBCL patients compared to CD19 CAR‐T therapy. Moreover, Ghorashian et al. [45] have analysed the CD19/22 co‐transduction model on twelve R/R B‐ALL patients and within a median follow‐up of 8.7 months, no cases of relapse by antigen‐negative escape were observed compared to their previous study where 5 of 14 patients relapsed with CD19‐negative disease within 7 months after infusion, suggesting dual targeting may have prevented antigen‐negative relapse. The underpinning mechanism may be that CD22 and other B cell markers are still expressed on CD19‐negative tumour cells, making the combinational targeting of CD19 and other tumour‐associated antigens a feasible and superior solution compared to single targeting of CD19 in TP53‐disrupted B cell malignancies [1, 42, 46].

Of note, the majority dual targeting CAR‐T therapy contained in this article represents cocktail treatment, which means sequential infusion of CD19 and CD22 CAR‐T product. In addition, there are also elaborately manufactured co‐administration CD19/CD22‐BBz CAR‐T cell therapies in use currently (i.e., eight cases in Li, 2023 [32]), which have been actively examined in preclinical and clinical studies [47]. CAR‐T cell persistence is a prerequisite to assessing dual targeting. It is found that transduction of more complex vectors may impact T‐cell phenotype, expansion, or persistence, thus leading to efficacy limitation when incorporating co‐administration and co‐transduction models [48]. In summary, the optimal strategy remains undefined and more comparative assessments for clinical benefits are warranted.

There are some drawbacks in the article as well. Heterogeneity is not controlled other than stratification through study design. Despite huge efforts being devoted to retrieve associated articles, the amount of interested studies is rare; the small candidate cohort may affect results.

## 5 Conclusion

In this meta‐analysis, we compared the CAR‐T therapeutic outcomes in B cell malignancy patients bearing TP53m or not. The findings indicated that though the CR and ORR of wild type and TP53m groups were similar, the PFS and OS of B cell malignancy patients bearing TP53m were inferior to wild type patients receiving CAR‐T cell treatment in this cohort. Notably, the estimated CR, PFS and OS of both groups exhibit the same therapeutic effect via dual targeting CAR‐T therapy. In other words, the poor prognosis of TP53m patients may be overcome by a novel bi‐targeting CAR‐T mode. Hopefully, this conclusion may aid in guiding CAR‐T associated clinical decision of the TP53m population with blood cancer. But the observed outcomes may be influenced by confounding factors, such as adverse disease biology or prior treatment effects, rather than a direct impact on CAR‐T efficacy. Further mechanistic and prospective studies and larger cohorts are needed to clarify whether TP53 mutations independently affect CAR‐T cell function or if the association reflects broader disease aggressiveness. Validation in larger cohorts and prospective trials is expected to explore and improve the outcome of CAR‐T efficacy in TP53m patients.

## Author Contributions

**Wenxin Qi:** formal analysis (lead), methodology (lead), resources (lead), resources (lead), software (equal), software (equal), writing – original draft (lead), writing – original draft (lead). **Weilong Zhang:** data curation (equal), resources (equal), supervision (equal), visualization (equal), writing – review and editing (equal). **Yuqi Zhang:** formal analysis (equal), methodology (equal), writing – original draft (equal), writing – review and editing (equal). **Xiaoyu Hao:** formal analysis (equal), resources (equal), writing – original draft (equal). **Ping Yang:** formal analysis (equal), funding acquisition (equal), writing – review and editing (equal). **Jing Wang:** funding acquisition (equal), methodology (equal). **Chaoling Wu:** writing – review and editing (equal). **Hongmei Jing:** funding acquisition (equal), project administration (equal), resources (equal), writing – review and editing (equal).

## Ethics Statement

The authors have nothing to report.

## Conflicts of Interest

The authors declare no conflicts of interest.

## Supporting information


**Appendix S1:** jcmm70818‐sup‐0001‐Supinfo.pdf.

## Data Availability

The datasets generated and analysed during this study are all available in the PubMed and Embase.

## References

[1] J. Wei , M. Xiao , Z. Mao , et al., “Outcome of Aggressive B‐Cell Lymphoma With TP53 Alterations Administered With CAR T‐Cell Cocktail Alone or in Combination With ASCT,” Signal Transduction and Targeted Therapy 7, no. 1 (2022): 101. 35399106 10.1038/s41392-022-00924-0 PMC8995369

[2] C. Gisselbrecht , B. Glass , N. Mounier , et al., “Salvage Regimens With Autologous Transplantation for Relapsed Large B‐Cell Lymphoma in the Rituximab Era,” Journal of Clinical Oncology 28, no. 27 (2010): 4184–4190. 20660832 10.1200/JCO.2010.28.1618 PMC3664033

[3] L. H. Sehn and G. Salles , “Diffuse Large B‐Cell Lymphoma,” New England Journal of Medicine 384, no. 9 (2021): 842–858. 33657296 10.1056/NEJMra2027612 PMC8377611

[4] R. C. Larson and M. V. Maus , “Recent Advances and Discoveries in the Mechanisms and Functions of CAR T Cells,” Nature Reviews. Cancer 21, no. 3 (2021): 145–161. 33483715 10.1038/s41568-020-00323-z PMC8353572

[5] T. Gu , M. Zhu , H. Huang , and Y. Hu , “Relapse After CAR‐T Cell Therapy in B‐Cell Malignancies: Challenges and Future Approaches,” Journal of Zhejiang University. Science. B 23, no. 10 (2022): 793–811. 36226535 10.1631/jzus.B2200256 PMC9561408

[6] C. Jacobson , F. L. Locke , A. Ghobadi , et al., “Long‐Term (≥4 Year and ≥5 Year) Overall Survival (OS) by 12‐ and 24‐Month Event‐Free Survival (EFS): An Updated Analysis of ZUMA‐1, the Pivotal Study of Axicabtagene Ciloleucel (Axi‐Cel) in Patients (Pts) With Refractory Large B‐Cell Lymphoma (LBCL),” Blood 138 (2021): 1764.

[7] M. C. Pasquini , Z. H. Hu , K. Curran , et al., “Real‐World Evidence of Tisagenlecleucel for Pediatric Acute Lymphoblastic Leukemia and Non‐Hodgkin Lymphoma,” Blood Advances 4, no. 21 (2020): 5414–5424. 33147337 10.1182/bloodadvances.2020003092 PMC7656920

[8] R. G. Majzner , S. P. Rietberg , E. Sotillo , et al., “Tuning the Antigen Density Requirement for CAR T‐Cell Activity,” Cancer Discovery 10, no. 5 (2020): 702–723. 32193224 10.1158/2159-8290.CD-19-0945 PMC7939454

[9] J. Y. Spiegel , S. Patel , L. Muffly , et al., “CAR T Cells With Dual Targeting of CD19 and CD22 in Adult Patients With Recurrent or Refractory B Cell Malignancies: A Phase 1 Trial,” Nature Medicine 27, no. 8 (2021): 1419–1431. 10.1038/s41591-021-01436-0 PMC8363505 34312556

[10] A. L. Garfall , A. D. Cohen , S. P. Susanibar‐Adaniya , et al., “Anti‐BCMA/CD19 CAR T Cells With Early Immunomodulatory Maintenance for Multiple Myeloma Responding to Initial or Later‐Line Therapy,” Blood Cancer Discovery 4, no. 2 (2023): 118–133. 36413381 10.1158/2643-3230.BCD-22-0074 PMC9975770

[11] Y. Wang , Y. Yang , R. Hong , et al., “A Retrospective Comparison of CD19 Single and CD19/CD22 Bispecific Targeted Chimeric Antigen Receptor T Cell Therapy in Patients With Relapsed/Refractory Acute Lymphoblastic Leukemia,” Blood Cancer Journal 10, no. 10 (2020): 105. 33077713 10.1038/s41408-020-00371-6 PMC7572410

[12] K. Hirabayashi , H. Du , Y. Xu , et al., “Dual Targeting CAR‐T Cells With Optimal Costimulation and Metabolic Fitness Enhance Antitumor Activity and Prevent Escape in Solid Tumors,” Nature Cancer 2, no. 9 (2021): 904–918. 34746799 10.1038/s43018-021-00244-2 PMC8570569

[13] C. A. Ramos , R. Rouce , C. S. Robertson , et al., “In Vivo Fate and Activity of Second‐ Versus Third‐Generation CD19‐Specific CAR‐T Cells in B Cell Non‐Hodgkin's Lymphomas,” Molecular Therapy 26, no. 12 (2018): 2727–2737. 30309819 10.1016/j.ymthe.2018.09.009 PMC6277484

[14] S. Negrini , V. G. Gorgoulis , and T. D. Halazonetis , “Genomic Instability–An Evolving Hallmark of Cancer,” Nature Reviews. Molecular Cell Biology 11, no. 3 (2010): 220–228. 20177397 10.1038/nrm2858

[15] A. O. Giacomelli , X. Yang , R. E. Lintner , et al., “Mutational Processes Shape the Landscape of TP53 Mutations in Human Cancer,” Nature Genetics 50, no. 10 (2018): 1381–1387. 30224644 10.1038/s41588-018-0204-y PMC6168352

[16] Y. Liu , Z. Su , O. Tavana , and W. Gu , “Understanding the Complexity of p53 in a New Era of Tumor Suppression,” Cancer Cell 42, no. 6 (2024): 946–967. 38729160 10.1016/j.ccell.2024.04.009 PMC11190820

[17] A. Chiappella , F. Diop , C. Agostinelli , et al., “Prognostic Impact of TP53 Mutation in Newly Diagnosed Diffuse Large B‐Cell Lymphoma Patients Treated in the FIL‐DLCL04 Trial,” British Journal of Haematology 196, no. 5 (2022): 1184–1193. 34951009 10.1111/bjh.17971

[18] T. Erazo , C. M. Evans , D. Zakheim , et al., “TP53 Mutations and RNA‐Binding Protein MUSASHI‐2 Drive Resistance to PRMT5‐Targeted Therapy in B‐Cell Lymphoma,” Nature Communications 13, no. 1 (2022): 5676. 10.1038/s41467-022-33137-8 PMC9515221 36167829

[19] T. X. Lu , K. H. Young , W. Xu , and J. Y. Li , “TP53 Dysfunction in Diffuse Large B‐Cell Lymphoma,” Critical Reviews in Oncology/Hematology 97 (2016): 47–55. 26315382 10.1016/j.critrevonc.2015.08.006

[20] J. Malcikova , S. Pavlova , P. Baliakas , et al., “ERIC Recommendations for TP53 Mutation Analysis in Chronic Lymphocytic Leukemia‐2024 Update,” Leukemia 38, no. 7 (2024): 1455–1468. 38755420 10.1038/s41375-024-02267-x PMC11217004

[21] T. Zenz , M. Kreuz , M. Fuge , et al., “TP53 Mutation and Survival in Aggressive B Cell Lymphoma,” International Journal of Cancer 141, no. 7 (2017): 1381–1388. 28614910 10.1002/ijc.30838

[22] T. Zhang , Y. Lu , X. Liu , et al., “Comprehensive Analysis of TP53 Mutation Characteristics and Identification of Patients With Inferior Prognosis and Enhanced Immune Escape in Diffuse Large B‐Cell Lymphoma,” American Journal of Hematology 97, no. 1 (2022): E14–E17. 34710245 10.1002/ajh.26392

[23] K. H. Young , K. Leroy , M. B. Møller , et al., “Structural Profiles of TP53 Gene Mutations Predict Clinical Outcome in Diffuse Large B‐Cell Lymphoma: An International Collaborative Study,” Blood 112, no. 8 (2008): 3088–3098. 18559976 10.1182/blood-2008-01-129783 PMC2569165

[24] Z. Y. Xu‐Monette , L. Wu , C. Visco , et al., “Mutational Profile and Prognostic Significance of TP53 in Diffuse Large B‐Cell Lymphoma Patients Treated With R‐CHOP: Report From an International DLBCL Rituximab‐CHOP Consortium Program Study,” Blood 120, no. 19 (2012): 3986–3996. 22955915 10.1182/blood-2012-05-433334 PMC3496956

[25] H. Shi , P. Zheng , R. Liu , et al., “Genetic Landscapes and Curative Effect of CAR T‐Cell Immunotherapy in Patients With Relapsed or Refractory DLBCL,” Blood Advances 7, no. 6 (2023): 1070–1075. 35901280 10.1182/bloodadvances.2021006845 PMC10034568

[26] N. Wang , X. Hu , W. Cao , et al., “Efficacy and Safety of CAR19/22 T‐Cell Cocktail Therapy in Patients With Refractory/Relapsed B‐Cell Malignancies,” Blood 135, no. 1 (2020): 17–27. 31697824 10.1182/blood.2019000017

[27] R. Shouval , A. Alarcon Tomas , J. A. Fein , et al., “Impact of TP53 Genomic Alterations in Large B‐Cell Lymphoma Treated With CD19‐Chimeric Antigen Receptor T‐Cell Therapy,” Journal of Clinical Oncology 40, no. 4 (2022): 369–381. 34860572 10.1200/JCO.21.02143 PMC8797602

[28] Y. Wang , P. Jain , F. L. Locke , et al., “Brexucabtagene Autoleucel for Relapsed or Refractory Mantle Cell Lymphoma in Standard‐Of‐Care Practice: Results From the US Lymphoma CAR T Consortium,” Journal of Clinical Oncology 41, no. 14 (2023): 2594–2606. 36753699 10.1200/JCO.22.01797 PMC10489553

[29] N. Gökbuget , N. Boissel , S. Chiaretti , et al., “Diagnosis, Prognostic Factors, and Assessment of ALL in Adults: 2024 ELN Recommendations From a European Expert Panel,” Blood 143, no. 19 (2024): 1891–1902. 38295337 10.1182/blood.2023020794

[30] B. D. Cheson , S. Ansell , L. Schwartz , et al., “Refinement of the Lugano Classification Lymphoma Response Criteria in the Era of Immunomodulatory Therapy,” Blood 128, no. 21 (2016): 2489–2496. 27574190 10.1182/blood-2016-05-718528

[31] J. F. Tierney , L. A. Stewart , D. Ghersi , S. Burdett , and M. R. Sydes , “Practical Methods for Incorporating Summary Time‐To‐Event Data Into Meta‐Analysis,” Trials 8 (2007): 16. 17555582 10.1186/1745-6215-8-16 PMC1920534

[32] Y. Li , Y. Liu , K. Yang , et al., “Impact of ARID1A and TP53 Mutations in Pediatric Refractory or Relapsed Mature B‐Cell Lymphoma Treated With CAR‐T Cell Therapy,” Cancer Cell International 23, no. 1 (2023): 281. 37981695 10.1186/s12935-023-03122-2 PMC10657579

[33] A. Minson , N. Hamad , C. Y. Cheah , et al., “CAR T Cells and Time‐Limited Ibrutinib as Treatment for Relapsed/Refractory Mantle Cell Lymphoma: The Phase 2 TARMAC Study,” Blood 143, no. 8 (2024): 673–684. 37883795 10.1182/blood.2023021306

[34] J. Pan , Y. Tan , B. Deng , et al., “Frequent Occurrence of CD19‐Negative Relapse After CD19 CAR T and Consolidation Therapy in 14 TP53‐Mutated r/r B‐ALL Children,” Leukemia 34, no. 12 (2020): 3382–3387. 32346068 10.1038/s41375-020-0831-z

[35] M. Wang , J. Munoz , A. Goy , et al., “Three‐Year Follow‐Up of KTE‐X19 in Patients With Relapsed/Refractory Mantle Cell Lymphoma, Including High‐Risk Subgroups, in the ZUMA‐2 Study,” Journal of Clinical Oncology 41, no. 3 (2023): 555–567. 35658525 10.1200/JCO.21.02370 PMC9870225

[36] X. Zhang , J. Yang , J. Li , et al., “Factors Associated With Treatment Response to CD19 CAR‐T Therapy Among a Large Cohort of B Cell Acute Lymphoblastic Leukemia,” Cancer Immunology, Immunotherapy 71, no. 3 (2022): 689–703. 34365516 10.1007/s00262-021-03009-z PMC10991205

[37] D. J. Baker , Z. Arany , J. A. Baur , J. A. Epstein , and C. H. June , “CAR T Therapy Beyond Cancer: The Evolution of a Living Drug,” Nature 619, no. 7971 (2023): 707–715. 37495877 10.1038/s41586-023-06243-w PMC12522170

[38] T. Tix , M. Alhomoud , R. Shouval , et al., “Second Primary Malignancies After CAR T‐Cell Therapy: A Systematic Review and Meta‐Analysis of 5,517 Lymphoma and Myeloma Patients,” Clinical Cancer Research 30, no. 20 (2024): 4690–4700. 39256908 10.1158/1078-0432.CCR-24-1798 PMC11546643

[39] M. Abou‐El‐Enein , “The Fate(s) of CAR T‐Cell Therapy: Navigating the Risks of CAR+ T‐Cell Malignancy,” Blood Cancer Discovery 5, no. 4 (2024): 249–257. 38713831 10.1158/2643-3230.BCD-23-0272 PMC11215381

[40] S. Liu , X. Zhang , H. Dai , et al., “Which One Is Better for Refractory/Relapsed Acute B‐Cell Lymphoblastic Leukemia: Single‐Target (CD19) or Dual‐Target (Tandem or Sequential CD19/CD22) CAR T‐Cell Therapy?,” Blood Cancer Journal 13, no. 1 (2023): 60. 37095120 10.1038/s41408-023-00819-5 PMC10125987

[41] A. J. Walker , R. G. Majzner , L. Zhang , et al., “Tumor Antigen and Receptor Densities Regulate Efficacy of a Chimeric Antigen Receptor Targeting Anaplastic Lymphoma Kinase,” Molecular Therapy 25, no. 9 (2017): 2189–2201. 28676342 10.1016/j.ymthe.2017.06.008 PMC5589087

[42] M. Ruella , F. Korell , P. Porazzi , and M. V. Maus , “Mechanisms of Resistance to Chimeric Antigen Receptor‐T Cells in Haematological Malignancies,” Nature Reviews. Drug Discovery 22, no. 12 (2023): 976–995. 37907724 10.1038/s41573-023-00807-1 PMC10965011

[43] W. Li , L. Ding , W. Shi , et al., “Safety and Efficacy of Co‐Administration of CD19 and CD22 CAR‐T Cells in Children With B‐ALL Relapse After CD19 CAR‐T Therapy,” Journal of Translational Medicine 21, no. 1 (2023): 213. 36949487 10.1186/s12967-023-04019-4 PMC10031882

[44] W. Sang , M. Shi , J. Yang , et al., “Phase II Trial of Co‐Administration of CD19‐ and CD20‐Targeted Chimeric Antigen Receptor T Cells for Relapsed and Refractory Diffuse Large B Cell Lymphoma,” Cancer Medicine 9, no. 16 (2020): 5827–5838. 32608579 10.1002/cam4.3259 PMC7433814

[45] S. Ghorashian , G. Lucchini , R. Richardson , et al., “CD19/CD22 Targeting With Cotransduced CAR T Cells to Prevent Antigen‐Negative Relapse After CAR T‐Cell Therapy for B‐Cell ALL,” Blood 143, no. 2 (2024): 118–123. 37647647 10.1182/blood.2023020621

[46] T. J. Fry , N. N. Shah , R. J. Orentas , et al., “CD22‐Targeted CAR T Cells Induce Remission in B‐ALL That Is Naive or Resistant to CD19‐Targeted CAR Immunotherapy,” Nature Medicine 24, no. 1 (2018): 20–28. 10.1038/nm.4441 PMC5774642 29155426

[47] S. Cordoba , S. Onuoha , S. Thomas , et al., “CAR T Cells With Dual Targeting of CD19 and CD22 in Pediatric and Young Adult Patients With Relapsed or Refractory B Cell Acute Lymphoblastic Leukemia: A Phase 1 Trial,” Nature Medicine 27, no. 10 (2021): 1797–1805. 10.1038/s41591-021-01497-1 PMC8516648 34642489

[48] H. Shalabi , H. Qin , A. Su , et al., “CD19/22 CAR T Cells in Children and Young Adults With B‐ALL: Phase 1 Results and Development of a Novel Bicistronic CAR,” Blood 140, no. 5 (2022): 451–463. 35605184 10.1182/blood.2022015795 PMC9353146

