# Supplementary material for: Outcomes of Wild Type and TP53‐Mutated B Cell Malignancy Patients Receiving CAR‐T Cell Therapy: A Systematic Review and Meta‐Analysis
Source: J Cell Mol Med. 2025 Sep 22;29(18):e70818. doi: 10.1111/jcmm.70818 (PMC12454170; doi:10.1111/jcmm.70818)
Supplement: Supplementary file 1 — Appendix S1: jcmm70818‐sup‐0001‐Supinfo.pdf. [file JCMM-29-e70818-s001.pdf]

Table S1. Inclusion criteria

Component	Criteria
Population	Patients with B cell malignancy with/without TP53 mutation
Intervention	Receiving CAR-T therapy
Outcomes	Primary outcomes:<br>Complete remission (CR)<br>Any PR/SD/PD<br>Median overall survival<br>Progression-free survival
Study design	Randomized controlled trials<br>Single-arm trials<br>Prospective observational studies<br>Retrospective studies<br>Meeting reports

Table S2. Search strategy

	Query	Result
MEDLINE	(((lymphoma[Title/Abstract]) OR (leukemia[Title/Abstract])) AND (CAR-T)) AND ((((((CAR-T) AND (TP53 mutation)) OR ((CAR-T) AND (survival analysis))) OR ((CAR-T) AND (Complete remission))) OR ((CAR-T) AND (ORR))) OR ((CAR-T) AND (Progression-free survival))) OR ((CAR-T) AND (OS))) AND ((humans[Filter]) AND (english[Filter]))	824
	('chimeric antigen receptor immunotherapy'/exp OR 'chimeric antigen receptor immunotherapy') AND ('lymphoma'/exp OR 'lymphoma' OR 'leukemia'/exp OR 'leukemia') AND (('chimeric antigen receptor immunotherapy'/exp OR 'chimeric antigen receptor immunotherapy') AND ('complete remission'/exp OR 'complete remission') OR (('chimeric antigen receptor immunotherapy'/exp OR 'chimeric antigen receptor immunotherapy') AND ('survival analysis'/exp OR 'survival analysis')) OR (('chimeric antigen receptor immunotherapy'/exp OR 'chimeric antigen	

EMBASE	receptor immunotherapy') AND ('progression-free survival'/exp OR 'progression-free survival' OR (('progression free'/exp OR 'progression free') AND ('survival'/exp OR survival)))) OR (('chimeric antigen receptor immunotherapy'/exp OR 'chimeric antigen receptor immunotherapy') AND ('tp53 mutation'/exp OR 'tp53 mutation')) OR (('chimeric antigen receptor immunotherapy'/exp OR 'chimeric antigen receptor immunotherapy') AND 'overall survival') OR (('chimeric antigen receptor immunotherapy'/exp OR 'chimeric antigen receptor immunotherapy') AND 'overall response rate')) AND [humans]/lim AND [english]/lim	3007

Table S3. Newcastle-Ottawa scale assessment for included studies

Study name	Selection	Comparability	Outcome
Shouval,2022	***	**	**
Hui Shi,2023	**	**	**
Yucai Wang,2023	***	**	**
Li,2023	***	**	*
Pan,2022	**	**	*
Zhang,2022	**	*	**

Table S4. The Joanna-Brigg's Institute assessment of study bias for included studies

Author	Year	Criterion 1: Inclusion criteria	Criterion 2: Measurement of wild type and TP53 mutation outcome	Criterion 3: Identification of wild type and TP54 mutation outcome	Criterion 4: Consecutive inclusion	Criterion 5: Complete inclusion	Criterion 6: Demographics	Criterion 7: Clinical information	Criterion 8: Follow-up results	Criterion 10: Apropriate statistical analysis
Minson	2024	Yes	Yes	Yes	Yes	No	Yes	Yes	Yes	Yes
Wei	2022	Yes	Yes	Yes	Yes	Yes	Yes	Yes	Yes	Yes
Michael Wang	2023	Yes	Yes	Yes	Yes	Yes	Yes	Yes	Yes	Yes
Na Wang	2020	Yes	Yes	Yes	Yes	No	Yes	Yes	Yes	Yes
Na Wang*	2020	Yes	Yes	Yes	Yes	Yes	Yes	Yes	Yes	Yes

Na Wang and Na Wang* are two studies from one publication.

Figure S1. Flow diagram

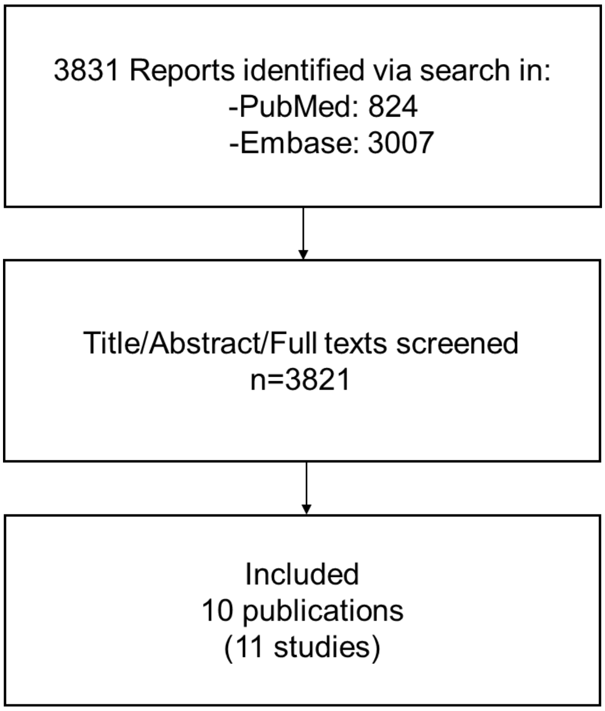

Figure S2.

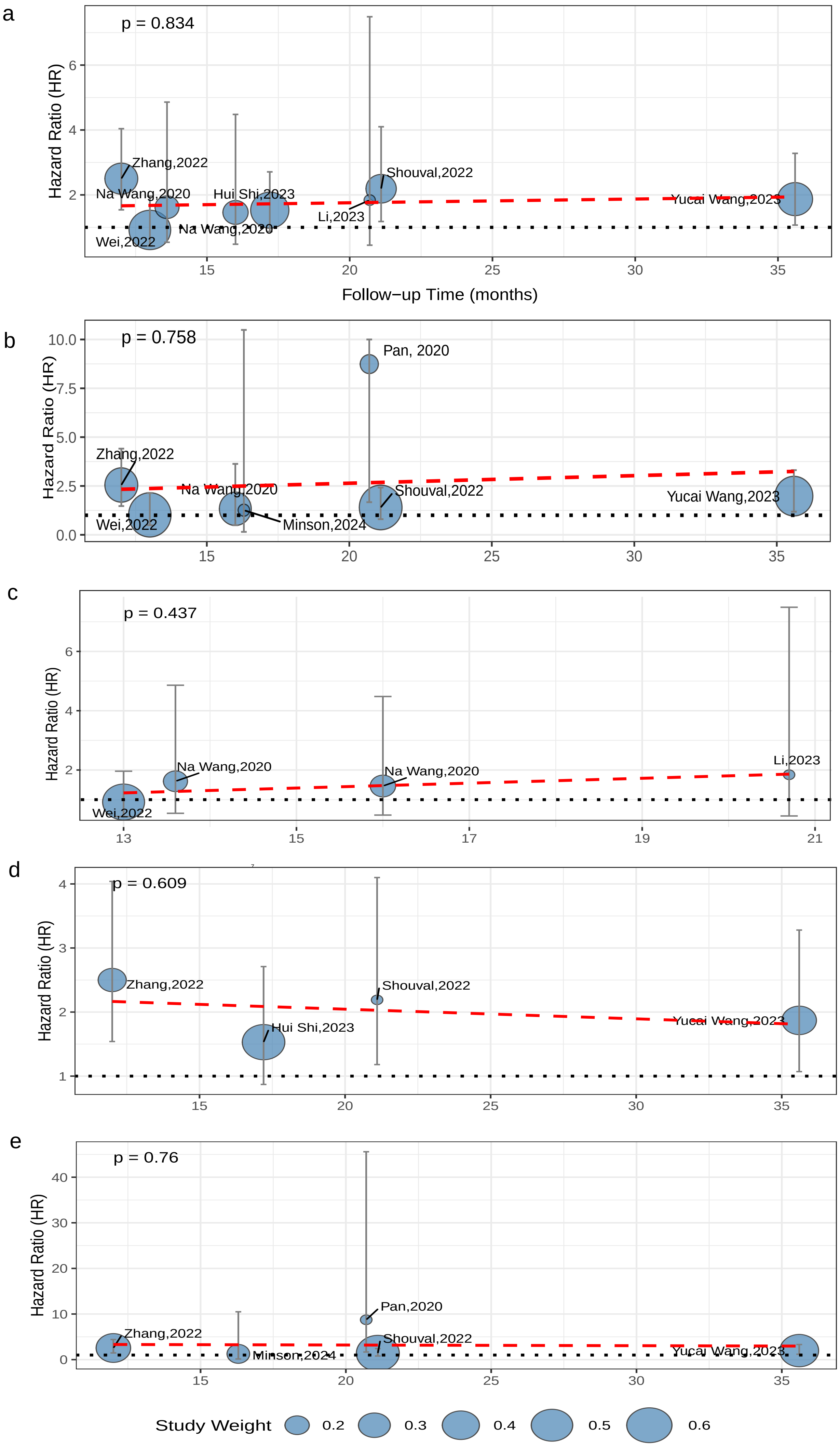
a
p = 0.834
Hazard Ratio (HR)
Zhang,2022
Na Wang,2020
Hui Shi,2023
Shouval,2022
Li,2023
Na Wang,2020
Wei,2022
Yucai Wang,2023
Follow−up Time (months)
b
p = 0.758
Hazard Ratio (HR)
Pan, 2020
Zhang,2022
Na Wang,2020
Shouval,2022
Yucai Wang,2023
Wei,2022
Minson,2024
c
p = 0.437
Hazard Ratio (HR)
Na Wang,2020
Na Wang,2020
Li,2023
Wei,2022
d
p = 0.609
Hazard Ratio (HR)
Zhang,2022
Shouval,2022
Hui Shi,2023
Yucai Wang,2023
e
p = 0.76
Hazard Ratio (HR)
Pan,2020
Zhang,2022
Shouval,2022
Minson,2024
Yucai Wang,2023
Study Weight
0.2
0.3
0.4
0.5
0.6

Figure S3.

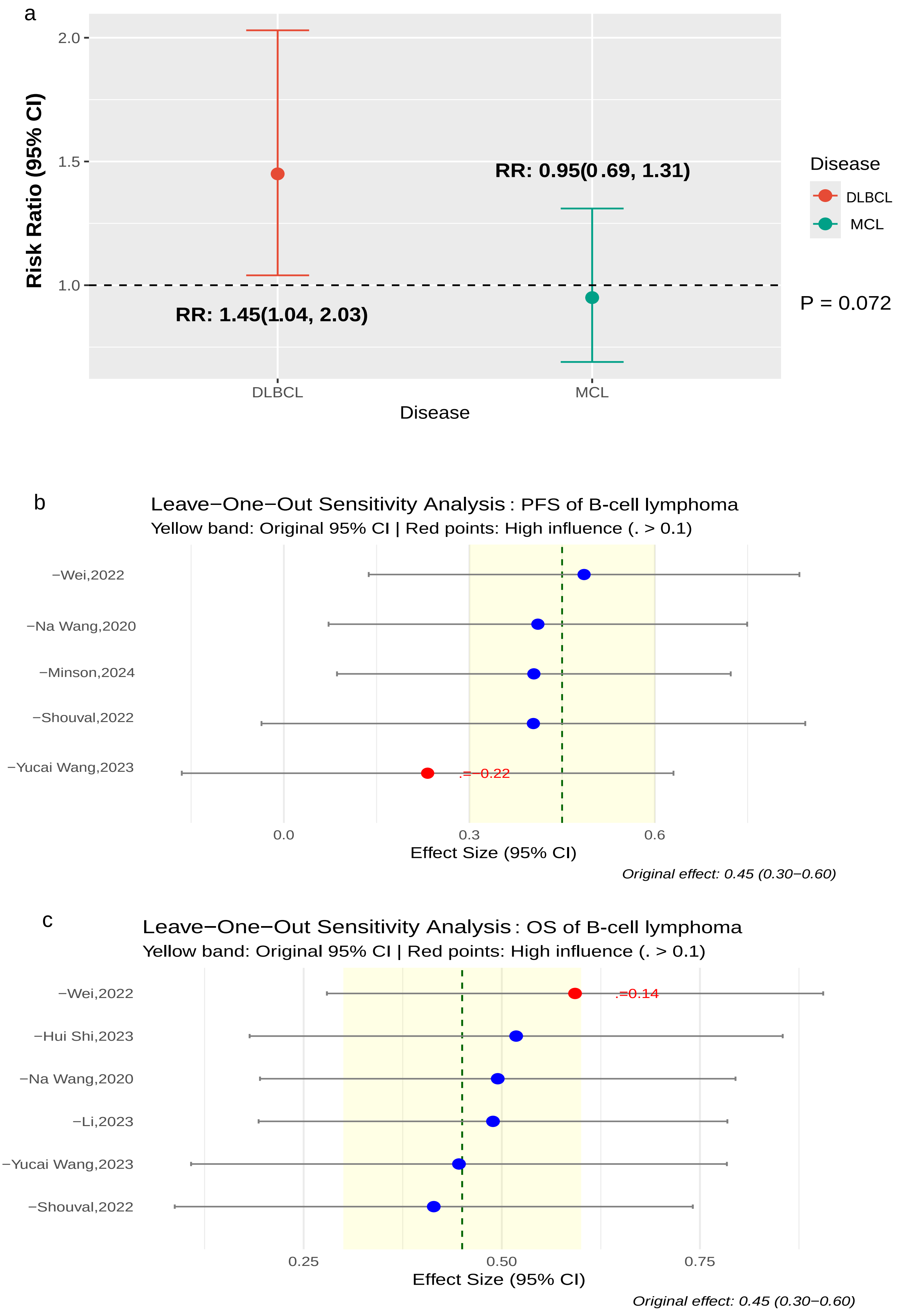
a
Risk Ratio (95% CI)
2.0
1.5
1.0
RR: 0.95(0.69, 1.31)
RR: 1.45(1.04, 2.03)
DLBCL
MCL
Disease
Disease
DLBCL
MCL
P = 0.072
b
Leave−One−Out Sensitivity Analysis : PFS of B-cell lymphoma
Yellow band: Original 95% CI | Red points: High influence (. > 0.1)
−Wei,2022
−Na Wang,2020
−Minson,2024
−Shouval,2022
−Yucai Wang,2023
.=−0.22
0.0
0.3
0.6
Effect Size (95% CI)
Original effect: 0.45 (0.30−0.60)
c
Leave−One−Out Sensitivity Analysis : OS of B-cell lymphoma
Yellow band: Original 95% CI | Red points: High influence (. > 0.1)
−Wei,2022
−Hui Shi,2023
−Na Wang,2020
−Li,2023
−Yucai Wang,2023
−Shouval,2022
.=0.14
0.25
0.50
0.75
Effect Size (95% CI)
Original effect: 0.45 (0.30−0.60)

Figure S4.

a

CAR-T	Study name	Disease	Treatment	Wild type		TP53m		Weight	Risk Ratio(95%CI)
				Size	CR	Size	CR		
Dual targeting	Wei,2022	DLBCL	CD19/22	34	50.0%	31	45.2%	100.0%	RR:1.11(0.66-1.55)
	Na Wang,2020	B-ALL	CD19/22	40	92.5%	11	100.0%	100.0%	RR:0.95(0.82-1.11)
Single targeting	Zhang,2022	B-ALL	CD19	221	93%	33	72.7%	100.0%	RR:1.28(1.04-1.58)
	Hui Shi,2023	DLBCL	CD19	40	52.5%	40	32.5%	51.3%	RR:1.62(0.95-2.76)
	Shouval,2022	DLBCL	CD19	52	64%	30	33.3%	46.7%	RR:1.90(1.10-3.29)
	Total				59.0%		32.9%	100.0%	RR:1.75(1.19-2.56)
	Heterogeneity: Chi^2 = 0.17, df = 1(P = 0.68); I^2 = 0%								
	Test for overall effect: Z = 1.19($P < 0.001$)								
	Minson,2024	MCL	CD19	12	75%	8	87.5%	12.4%	RR:0.86(0.56-1.30)
	Yucai Wang,2023	MCL	CD19	65	88.0%	61	72.0%	64.6%	RR:1.22(1.01-1.46)
	Michael,2023	MCL	CD19	30	70%	6	100.0%	23.0%	RR:0.75(0.55-1.02)
	Total				81.5%		75.9%	100.0%	RR:1.04(0.90-1.21)
	Heterogeneity: Chi^2 = 7.97, df = 2(P = 0.02); I^2 = 75%								
	Test for overall effect: Z = 2.87(P = 0.56)								

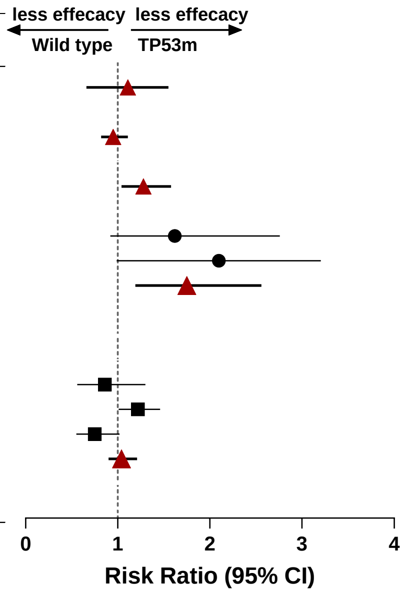

b

CAR-T	Study name	Disease	Treatment	Wild type		TP53m		Weight	Hazard Ratio(95%CI)
				Size	PFS	Size	PFS		
Dual targeting	Wei,2022	DLBCL	CD19/22	34	60%(1y)	31	56%(1y)	64.9%	HR:1.02(0.49-2.14)
	Na Wang, 2020	DLBCL	CD19/22	28	42%(1y)	10	50%(1y)	35.1%	HR:1.33(0.49-3.63)
	Total				52%(1y)		55%(1y)	100.0%	HR:1.12(0.62-2.03)
	Heterogeneity: Chi^2 =0.17, df = 1(P = 0.68); I^2 = 0%								
	Test for overall effect: Z = 0.38(P=0.70)								
Single targeting	Shouval,2022	DLBCL	CD19	52	21.1m	30	34%(1y)	100.0%	HR:1.40(0.80-2.42)
	Minson,2024	MCL	CD19	10	80%(1y)	8	75%(1y)	5.6%	HR:1.26(0.15-10.49)
	Yucai Wang,2023	MCL	CD19	65	63%(1y)	61	47%(1y)	94.4%	HR:1.98(1.18-3.31)
	Total				65%(1y)		50%(1y)	100.0%	HR:1.94(1.17–3.19)
	Heterogeneity: Chi^2 = 0.16, df = 1(P = 0.69); I^2 = 0%								
	Test for overall effect: Z = 2.57(P = 0.01)								
	Pan,2020	B-ALL	CD19	40	90%(1y)	14	39% (1y)	9.9%	HR:8.74(1.67-45.58)
	Zhang,2022	B-ALL	CD19	207	72%(1y)	24	48% (1y)	90.1%	HR:2.55(1.47-4.41)
	Total				74%(1y)		45%(1y)	100.0%	HR:2.88(1.71–4.85)
	Heterogeneity: Chi^2 = 1.92, df = 1(P = 0.17); I^2 = 0%								
	Test for overall effect: Z = 3.98($P < 0.001$)								

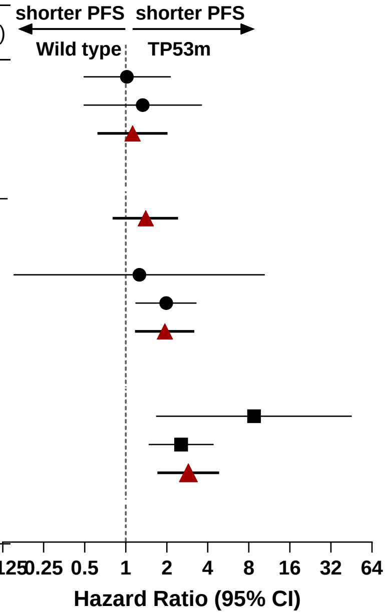

c

CAR-T	Study name	Disease	Treatmen	Wild type		TP53m		Weight	Hazard Ratio(95%CI)
				Size	OS	Size	OS		
Dual targeting	Na Wang,2020	B-ALL	CD19/22	40	60%(1y)	11	72%(1y)	100.0%	HR:1.46(0.48-4.48)
	Li,2023	B-NHL	CD19/22	10	74.5%(1y)	27	46.5%(1y)	100.0%	HR:1.84(0.45-7.49)
	Wei,2022	DLBCL	CD19/22	34	64%(1y)	31	64%(1y)	67.7%	HR:0.92(0.43-1.96)
	Na Wang,2020	DLBCL	CD19/22	28	53%(1y)	10	51%(1y)	32.3%	HR:1.62(0.54-4.86)
	Total				61%(1y)		59%(1y)		HR:1.10(0.59-2.06)
Heterogeneity: Chi^2 = 0.69, df = 1(P = 0.41); I^2 = 0%									
Test for overall effect: Z = 0.31(P = 0.76)									
Mono targeting	Zhang,2022	B-ALL	CD19	221	78%(1y)	33	55% (1y)	100.0%	HR:2.50(1.54–4.04)
	Yucai Wang,2023	MCL	CD19	65	NA	61	NA	100.0%	HR:1.87(1.07-3.28)
	Hui Shi,2023	DLBCL	CD19	40	65.6%(1y)	40	50%(1y)	54.6%	HR:1.53(0.87-2.71)
	Shouval,2022	DLBCL	CD19	52	76%(1y)	30	44% (1y)	45.4%	HR:2.19(1.18-4.10)
	Total				71%(1y)		47%(1y)		HR:1.80(1.18–2.74)
Heterogeneity: Chi^2 = 0.7, df = 1(P = 0.40); I^2 = 0%									
Test for overall effect: Z = 2.75(P = 0.01)									

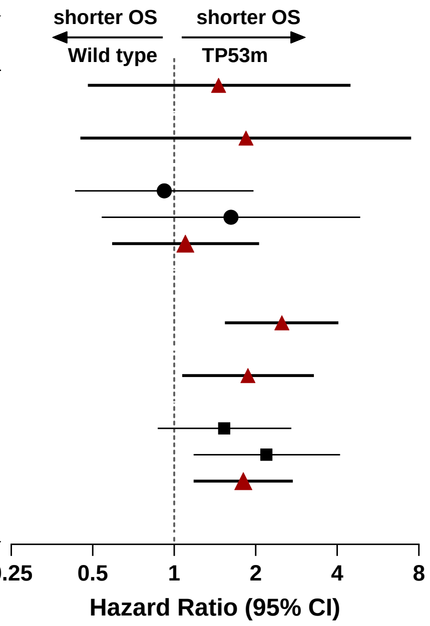

Supplementary Figure legend:

Supplementary Figure 1. Flow diagram of study selection

Supplementary Figure 2. Restricted Maximum Likelihood(REML) analysis of follow-up time and HR

(a) HR by follow-up time (OS of B cell lymphoma)

(b) HR by follow-up time (PFS of B cell lymphoma)

(c) HR by follow-up time (OS of dual-targeting CAR-T therapy)

(d) HR by follow-up time (OS of single-targeting CAR-T therapy)

(e) HR by follow-up time (PFS of single-targeting CAR-T therapy)

Supplementary Figure 3. Sensitivity analysis

(a) Disease-specific subgroup analysis of CR in Figure 3.

(b) Leave-one-out sensitivity analysis of PFS of B cell lymphoma in Figure 3.

(c) Leave-one-out sensitivity analysis of OS of B cell lymphoma in Figure 3.

Supplementary Figure 4.a-c. Disease-specific subgroup analysis of double-targeting and single-targeting CAR-T therapy in Figure 4.
